# Splicing landscape of the eight collaborative cross founder strains

**DOI:** 10.1186/s12864-015-1267-0

**Published:** 2015-02-05

**Authors:** Christina L Zheng, Beth Wilmot, Nicole AR Walter, Denesa Oberbeck, Sunita Kawane, Robert P Searles, Shannon K McWeeney, Robert Hitzemann

**Affiliations:** Department of Medical Informatics and Clinical Epidemiology, Division of Bioinformatics and Computational Biology, Oregon Health & Science University, Portland, Oregon USA; Knight Cancer Institute, Oregon Health & Science University, Portland, Oregon USA; Oregon Clinical and Translational Research Institute, Oregon Health & Science University, Portland, Oregon USA; Department of Behavioral Neuroscience, Oregon Health & Science University, Portland, Oregon USA; Integrated Genomics Laboratory, Oregon Health & Science University, Portland, Oregon USA; Department of Public Health and Preventative Medicine, Division of Biostatistics, Oregon Health & Science University, Portland, Oregon USA; Portland Alcohol Research Center, Oregon Health & Science University, Portland, Oregon USA; Veterans Affairs Research Service, Portland, OR USA

**Keywords:** Collaborative Cross, Splicing landscape, Strain specific splicing

## Abstract

**Background:**

The Collaborative Cross (CC) is a large panel of genetically diverse recombinant inbred mouse strains specifically designed to provide a systems genetics resource for the study of complex traits. In part, the utility of the CC stems from the extensive genome-wide annotations of founder strain sequence and structural variation. Still missing, however, are transcriptome-specific annotations of the CC founder strains that could further enhance the utility of this resource.

**Results:**

We provide a comprehensive survey of the splicing landscape of the 8 CC founder strains by leveraging the high level of alternative splicing within the brain. Using deep transcriptome sequencing, we found that a majority of the splicing landscape is conserved among the 8 strains, with ~65% of junctions being shared by at least 2 strains. We, however, found a large number of potential strain-specific splicing events as well, with an average of ~3000 and ~500 with ≥3 and ≥10 sequence read coverage, respectively, within each strain. To better understand strain-specific splicing within the CC founder strains, we defined criteria for and identified high-confidence strain-specific splicing events. These splicing events were defined as exon-exon junctions 1) found within only one strain, 2) with a read coverage ≥10, and 3) defined by a canonical splice site. With these criteria, a total of 1509 high-confidence strain-specific splicing events were identified, with the majority found within two of the wild-derived strains, CAST and PWK. Strikingly, the overwhelming majority, 94%, of these strain-specific splicing events are not yet annotated. Strain-specific splicing was also located within genomic regions recently reported to be over- and under-represented within CC populations.

**Conclusions:**

Phenotypic characterization of CC populations is increasing; thus these results will not only aid in further elucidating the transcriptomic architecture of the individual CC founder strains, but they will also help in guiding the utilization of the CC populations in the study of complex traits. This report is also the first to establish guidelines in defining and identifying strain-specific splicing across different mouse strains.

**Electronic supplementary material:**

The online version of this article (doi:10.1186/s12864-015-1267-0) contains supplementary material, which is available to authorized users.

## Background

Due to their genetic and physiological similarities to humans, mice have developed into the premier mammalian model system for genetic research [1-6]. Mice naturally develop many diseases, such as cancer [7,8], osteoporosis [9,10] and diabetes [11]. For human diseases that do not naturally afflict mice, such as cystic fibrosis and Alzheimer’s disease, they can be induced through the manipulation of the mouse genome and environment [12,13]. Concerns over the limitations of mouse models have been raised [14,15], however, and reinforced by the poor concordance of some mouse studies and clinical trials. For example, the transcriptional response to various inflammatory insults (sepsis, trauma, burns and endotoxemia) appears to be quite different in mice and humans [16], raising the possibility that the mouse poorly models human inflammatory mechanisms. However, this conclusion was questioned on the basis of several key experimental details, including the fact that nearly all of the mouse data in these inflammation studies were collected in a single inbred strain, the C57BL/6 J [17,18]. Thus from a genetic perspective, the genetic diversity of inflammatory responses across *Mus musculus* was not fully captured.

Genetic diversity is a crucial component when assessing clinical relevance thus leading to the development of genetically diverse mouse resources such as the Collaborative Cross (CC) [19]. The eight-way breeding scheme of the CC, which included five classical inbred laboratory strains {C57BL/6 J (B6), 129S1/SvlmJ (129), A/J (AJ), NOD/ShiLtJ (NOD), and NZO/HILtJ (NZO)} and three wild-derived strains {CAST/EiJ (CAST), PWK/PhJ (PWK) and WSB/EiJ (WSB)}, was community-driven and designed specifically to minimize unpredictable genomic interactions between strains while optimizing the genomic contributions of all strains. Initial studies have reported promise for the utility of the CC mouse to aid in genetically dissecting complex traits, including albumin levels [20], hematological traits [21], susceptibility to periodontitis [22], and response to infection [23,24]. In part, the utility of the CC stems from genome-wide annotations of founder strain sequence and structural variations [25,26]. CC studies involving quantitative trait locus (QTL) analyses [27] and the generation of imputed genomes/transcriptomes [28] is heavily reliant on these annotations.

The addition of transcriptome-specific annotations for the CC founder strains would further enhance the utility of the CC. Studies on differential exon expression [29] and alternative exon usage [30] have been reported for selected mouse strains, as well as characterization of infection-related transcription in the lung in the 8 CC founders [31]. However a systematic characterization and comparison of the transcriptome structure of all 8 CC founder strains, at baseline, wherein transcriptome-specific information such as conserved and/or strain-specific splicing, transcript, or expression annotation, would add invaluable insight and utility to the CC. More detailed transcriptome-specific information for the individual CC founder strains may also provide additional insight into previously reported strain-specific phenotypes [32-34], improved imputed transcriptomes/genomes for individual strains and resultant crosses [28], and guidance for strain specific therapeutics models [35,36].

Here, we describe a comprehensive characterization of the splicing landscape of each of the CC founder strains at the resolution of individual exon-exon junctions. For this analysis, we utilized samples from the striatum, attempting to leverage the high level of alternative splicing within the brain [37]. Striatum-specific transcriptome information will also be an invaluable resource for region-specific transcriptome studies within the brain. Using RNA-seq, we found that the majority of the splicing landscape is conserved among the founder strains. However a large number of potential strain-specific splicing (SSS) events were also found, highlighting the unique transcriptome architecture of each strain. To further characterize and understand SSS, we defined and identified a set of high-confidence SSS events. Interestingly, the majority of these events were unannotated, with two of the wild-derived strains, CAST and PWK, showing the largest number of SSS events. SSS events were also found within recently reported over- and under-represented genomic regions of CC populations [38]. Collectively, these results will aid in further understanding and enhancing the utility of individual CC founder strains and all resultant mouse resources generated with these founder strains which include the CC, a HS population (HS-CC) [39] and the Diversity Outcross (DO) [40]. This large-scale assessment also allows us to provide guidance to the research community in defining and identifying SSS across different mouse strains.

## Methods

### Animal care

Three male mice from each of the 8 CC founder strains (C57BL/6 J, 129S1/SvlmJ, A/J, NOD/ShiLtJ, NZO/HILtJ, CAST/EiJ, PWK/PhJ, and WSB/EiJ) were obtained from The Jackson Laboratory (Sacramento, CA) and housed within the Portland Veterans Affairs Medical Center (PVAMC) Veterinary Medical Unit, an AAALAC-approved facility. Animals were housed by strain in polycarbonate or polysulfone cages with ecofresh bedding. Mice were fed standard rodent chow (Purina 5001, PMI Nutrition International, Brentwood, MO, USA). Food and water were available *ad libitum*. The rooms were maintained at 22 ± 1°C and on a 12:12 hour light:dark cycle (lights on at 0600). Animal use and care was approved by the Institutional Animal Care and Use Committee at the PVAMC and was in compliance with NIH and USDA guidelines.

### Sample preparation

At eight weeks old, mice were euthanized by cervical dislocation, and the brains were removed and immediately frozen on dry ice. Frozen brains were slightly thawed and dissected by hand using a razor blade under RNAse-free conditions. Using the optic chiasm as the caudal marker, a 2 mm coronal slice of brain tissue was isolated. Beginning at the medial ventral aspect of the striatum and recognizing that the striatum has a partial cone shape, the dissection moved dorsal 1 mm, followed by a cut to the lateral boundary of the striatum, with a final cut following the lateral-ventral boundary. The isolated tissue was immediately placed into 100 ml of Trizol (Invitrogen, Carlsbad, CA). On average, the isolated striatal tissue samples weighed 3 mg. RNA extraction was performed according the Trizol manufacturers’ protocol, followed by a sodium acetate precipitation to further remove any contaminants.

### Generation and QC of sequencing libraries

Sequencing libraries were constructed using the Illumina TruSeq RNAseq kit. Poly(A) + RNA was isolated from total RNA using oligo-dT coated magnetic beads. The recovered RNA was then chemically fragmented. First strand cDNA was generated using random hexamers as primers for reverse transcriptase. Following second strand synthesis, the ends of the double stranded fragments were repaired, and then a single “A” nucleotide was added to each end. Illumina adaptors were ligated to the cDNAs. Limited round PCR was used to amplify the material to yield the final library. Library concentration was determined using real-time PCR with primers complementary to the Illumina adaptors. Sample libraries were diluted and applied to an Illumina paired end flow cell at a concentration appropriate to generate about 180 million reads per lane. One sample was applied per lane and 100 cycle paired-end sequencing was used to generate base call files. Illumina’s CASAVA package was used to assemble the reads into standard FASTQ formatted data. FASTQC (http://www.bioinformatics.babraham.ac.uk/projects/fastqc/) was used to check the quality along the full lengths of the read in addition to assessing for the presence of sequence biases.

### Reference genome alignment and exon-exon junction detection

Paired-end sequenced reads were aligned to the B6 reference genome (mm9) using STARv2.3 [41] allowing for a maximum of 2 mismatches. Read pairs were required to map uniquely to the genome; thus, read pairs that did not map or mapped to multiple locations in the genome were not included in the analysis. To ensure a comprehensive detection of exon-exon junctions, a database of annotated exon-exon junctions (Ensembl version 66) was provided to STAR, a minimum distance was not enforced for the detection of neighboring junctions, and the same criteria (e.g., minimum read coverage) was used to identify both canonical and noncanonical spliced junctions. Both annotated and unannotated exon-exon junctions were reported.

### Comparison of splicing landscape across the 8 founder strains

Identified exon-exon junctions were compared across the 8 founder strains to identify junctions conserved across all strains. Hierarchical clustering, based on the Euclidean distance of the number of conserved junctions between each pair of strains, was used to visualize the splicing phylogeny across the 8 strains. Identified exon-exon junctions were also compared to annotated exon-exon junctions (Ensembl Version 66) to assess the percentage of annotated and unannotated splicing events.

### Experimental validation of strain-specific junctions

First strand cDNA was prepared from striatum total RNA (3 for each strain) using Life Technologies’ High Capacity cDNA Reverse Transcription Kit with random primers. PCR primers were designed to the strain-specific junctions and conserved junctions within the same gene using Primer3 [42] for primer design and standard Qiagen HotStar (Valencia, CA) PCR conditions: 95° 10 min; 35 cycles of: 95° 15 sec, 55° 1 min 15 sec, 72° 1 min 15 sec; final cycle of 72° 10 min. Primer sequences are listed in Additional file 1: Table S1.

## Results

In an effort to characterize the splicing landscape of the 8 CC founder strains, deep transcriptome sequencing was performed for each of the 8 strains. One RNA-Seq library from the striatum of 3 pooled animals for each strain was sequenced, using the Illumina HiSeq2000. Each library was run on one paired-end lane, resulting in each library ranging from 162 M-189 M 101 bp paired-end reads. RNA-seq reads for each strain were aligned to the mouse B6 reference genome (mm9) using STARv2.3 [41]. For all strains, ~88% of the read pairs mapped uniquely to the reference genome with ~30% mapping to exon-exon junctions. On average, ~5% of read pairs for each strain could not be mapped to the reference genome. Two of the wild-derived strains, CAST and PWK, had the highest percentages (7%) of unmapped reads, consistent with previous DNA studies [25]. Table 1 provides the library sizes and general alignment statistics for each library.

**Table 1:** **RNA-seq alignment statistics for the 8 collaborative cross founder strains**

**Strain**	**Total Read Pairs**	**Uniquely Mapped**	**Unmapped**	Spliced*
129	164920277	89.08%	5.08%	29.60%
AJ	169557445	89.87%	5.24%	26.95%
B6	172639054	88.71%	4.50%	29.70%
CAST	189948424	87.18%	7.72%	30.35%
NOD	178924375	88.70%	4.38%	30.22%
NZO	183452793	88.84%	4.50%	30.29%
PWK	175674996	86.92%	7.46%	30.40%
WSB	162484516	87.80%	4.65%	30.20%

*Proportion of uniquely mapped reads mapping across a spliced exon-exon junction.

### Identification of exon-exon junctions within the 8 CC founder strains

In an effort to obtain a more comprehensive set of exon-exon junctions for each strain, we used a database of annotated exon-exon junctions (Ensembl version 66) during the alignment of the RNA-seq reads, and we used non-stringent criteria to map individual reads across exon-exon junctions; these criteria included not imposing a minimum distance for neighboring exon-exon junctions and not requiring extra read support for non-canonical splice sites (see Methods section). Both annotated and unannotated exon-exon junctions were reported.

With these criteria, we found that ~98% of spliced-reads (reads that mapped across a spliced junction) mapped to previously annotated exon-exon junctions. Approximately 98% of spliced-reads also mapped to canonical GT/AG consensus splice sites (Table 2). This high mapping rate to previously annotated junctions and canonical splice sites increases our confidence in the identified exon-exon junctions, particularly in light of our more permissive junction identification criteria. Table 2 reports the alignment statistics of the spliced-reads for each strain.

**Table 2:** **Spliced-read alignment statistics for the 8 collaborative cross founder strains**

**Strain**	**Annotated Junctions**	**GT/AG**	**GC/AG**	**AT/AC**	**Non-Canonical**
129	98.56%	98.86%	0.83%	0.10%	0.21%
AJ	98.42%	98.81%	0.82%	0.11%	0.26%
B6	98.61%	98.93%	0.82%	0.10%	0.15%
CAST	98.40%	98.70%	0.83%	0.10%	0.37%
NOD	98.54%	98.88%	0.82%	0.11%	0.19%
NZO	98.54%	98.86%	0.83%	0.11%	0.20%
PWK	98.39%	98.73%	0.82%	0.11%	0.34%
WSB	98.49%	98.85%	0.82%	0.11%	0.22%

From our mapping, we identified an average of 370,000 exon-exon junctions within each strain. The majority of the identified junctions (~85%) in each strain were found to have the canonical GT/AG consensus splice site, with approximately 50% of the identified junctions being previously annotated. However these identified junctions represented the majority of all previously annotated exon-exon junctions: ~73% of annotated junctions were represented within each strain and 83% of annotated junctions were represented among all strains. Moreover, ~90% of junctions within each strain overlapped with an annotated gene (required to be on the same strand, when the strand was known). Table 3 reports the mapped exon-exon junction statistics for each strain.

**Table 3:** **Summary statistics for the identified exon-exon junctions for the 8 collaborative cross founder strains**

**Strain**	**Mapped Junctions**	**Annotated Junctions**	**GT/AG**	**GC/AG**	**AT/AC**	**Non-Canonical**	Overlap Gene*
129	361376	50.57%	87.80%	1.88%	0.21%	10.11%	91.80%
AJ	379320	47.18%	83.35%	2.19%	0.22%	14.24%	89.70%
B6	360792	50.05%	88.05%	1.80%	0.20%	9.95%	92.00%
CAST	380749	47.92%	85.25%	2.06%	0.21%	12.48%	91.57%
NOD	371622	48.83%	87.33%	1.92%	0.21%	10.54%	91.23%
NZO	381380	47.94%	87.10%	1.92%	0.19%	10.79%	91.04%
PWK	365628	48.75%	85.70%	2.00%	0.21%	12.09%	91.37%
WSB	360681	49.78%	87.30%	1.93%	0.22%	10.55%	91.62%

*Required to be on the same strand, when strand is known.

### The majority of the splicing landscape is conserved

A comprehensive comparison of all exon-exon junctions across the 8 founder strains found that the majority of the splicing landscape is conserved. Approximately 65% of all exon-exon junctions were found to be conserved in at least 2 strains. This percentage steadily increased as the read coverage for the exon-exon junctions increased. For junctions with ≥3 and ≥10 read coverage, the percentage of junctions conserved in at least 2 strains was ~86% and ~93%, respectively. A large percentage of exon-exon junctions were also conserved among all strains, particularly within the junctions with higher read coverage. Approximately 20%, 50% and 71% of junctions were conserved in all strains with ≥1, ≥3, and ≥10 read coverage, respectively. These data illustrate that the splicing landscape within the striatum, particularly among the highly expressed exon-exon junctions, is strongly conserved. It will be of great interest to investigate whether this level of conservation extends to other brain regions and other tissues.

### CAST and PWK have the most divergent transcriptome structure

**Figure 1:**
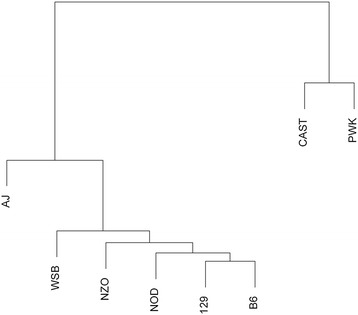
**Splicing phylogeny of the 8 founder strains.** Hierarchical clustering, based on the Euclidean distance of the number of conserved junctions between each pair of strains, visualizes the splicing phylogeny across the 8 strains. All identified exon-exon junctions from each strain were included. As expected, two of the wild-derived strains, CAST and PWK, show to have the most divergent transcriptome structures. Unexpectedly, the clustering of the classical laboratory strains and WSB, are different than that of previous clustering based on genomic data, suggesting different selective pressures on the transcriptomic and genomic levels.

A pair-wise comparison of all junctions between all strains showed that two of the wild-derived strains, CAST and PWK, were the most divergent (Figure 1). WSB, which could be considered a founder of the laboratory strains, showed less divergence. The observed divergence of the wild-derived strains at the transcriptome level is consistent with previous findings at the genomic level [43]. Interestingly, differences in the phylogenies observed among the classical laboratory strains in this study compared with Threadgill et al. (2011) [43] suggest that differential selective pressures may be acting at the transcriptome level versus the genome level [44].

### Protocol for the identification of high confidence strain-specific splicing events

In an effort to understand and characterize strain-related splicing differences, we set out to identify splicing events specific to each strain. The identification of SSS using RNA-seq is complicated by both experimental and analytical challenges thus the following protocol was used to identify a set of high confidence SSS events within each strain.

### Step 1: identification of potential strain-specific splicing events

The first step in identifying SSS events was to identify exon-exon junctions that were detected in only one strain. As expected, the number of strain-specific junctions decreased as read coverage increased (Table 4). An average of ~64,000 strain-specific junctions were identified for each strain when all junctions were considered; however for junctions with ≥3 or ≥10 read coverage, the numbers dropped to an average of approximately 3,000 and 500, respectively. The wild-derived strains, CAST and PWK, consistently demonstrated to have the highest percentages of potential strain-specific junctions.

**Table 4:** **Potential strain-specific junctions for each of the 8 collaborative cross founder strains based on a range of read coverage thresholds**

**Strain**	> = 1*	> = 3*	> = 10*
129	54863	1537	119
AJ	73998	2527	171
B6	54669	1038	67
CAST	80391	7610	1496
NOD	58343	1199	83
NZO	62475	1281	106
PWK	71756	6148	1228
WSB	56765	1908	303

*Read Coverage.

Due to a combination of potential sequencing errors and alignment artifacts, we caution that some of the identified strain specific exon-exon junctions, particularly those with low read coverage, may be false positives. Thus we label these strain-specific exon-exon junctions as potential SSS events.

### Step 2: determining minimum read coverage needed to detect a strain-specific splicing event

In an attempt to minimize the impact of potential sequencing errors on our identification of SSS events, the next task was to determine the minimum exon-exon junction read coverage needed to reliably detect an expressed splicing event. With this in mind, we chose a set of fourteen potential strain-specific exon-exon junctions to be experimentally validated. To assess our confidence in exon-exon junction with lower read coverage, we chose exon-exon junctions with a read coverage as low as 3. The following list shows the read coverage followed by the number of each class in parentheses: 3 (5), 4 (1), 6 (2), 13 (1), 15 (1), 20 (1), 23 (1), 24 (1), and 47 (1). PCR primers were designed for each potential strain-specific junction. Serving as a positive control, PCR primers were also designed for a neighboring exon-exon junction found to be conserved in all strains within the same gene.

**Figure 2:**
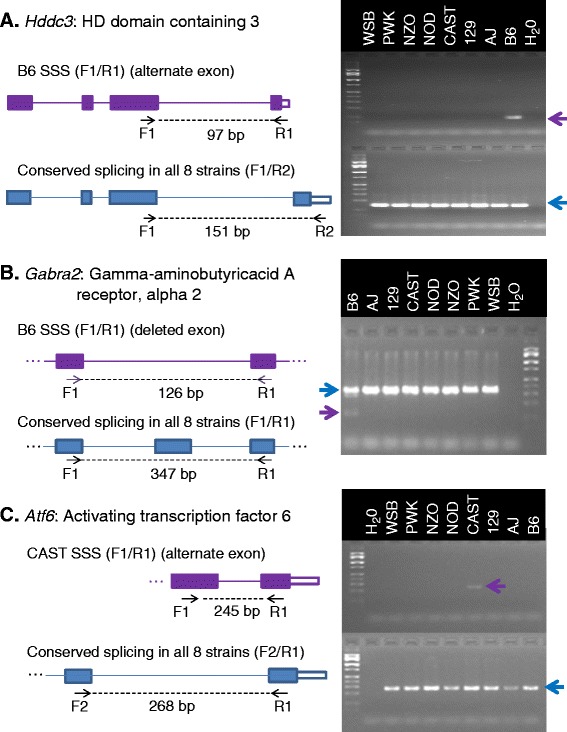
**Experimental validation of 3 strain-specific splicing events.** PCR validation, using striatum cDNA, of 3 SSS events. SSS events found within *Hddc3* **(A)**, *Gabra2* **(B)**, and *Atf6* **(C)** are depicted in purple. The B6 SSS event found within the *Hddc3* gene is supported with a read coverage of 20 and results in the use of an alternative last exon. The B6 SSS event found within the *Gabra2* gene is supported with a read coverage of 6 and results in a skipping of an exon. The CAST SSS event found within the *Atf6* gene is also supported by 6 reads. PCR products confirming each SSS event are indicated with purple arrows. A splicing event conserved within all 8 strains (blue), within each gene, served as a positive control. Blue arrows indicate the PCR products confirming each conserved splicing event. The length of the expected PCR product(s) from each forward (F) and reverse (R) primer are indicated. Water served as a negative control. A 100 bp ladder was loaded to confirm PCR product sizes.

All 14 exon-exon junctions were validated to exist; with 10 out of the 14 junctions validated as being strain-specific, resulting in a success rate greater than 70% for validated SSS events. Figure 2 depicts the experimental validation of 3 SSS events: one in the HD domain containing 3 (*Hdd3*) gene; one in the Gamma-aminobutyric acid A receptor, alpha 2 (*Gabra2*) gene; and one in Activating transcription factor 6 (*Atf6*) gene. The B6 SSS event found within the *Hddc3* gene is supported with a read coverage of 20 and results in the use of an alternative last exon. The B6 SSS event found within the *Gabra2* gene is supported with read coverage of 6 and results in a skipping of an exon. The CAST SSS event found within the *Atf6* gene is also supported by 6 reads. A full list of validated strain-specific junctions is available in Additional file 1: Table S1.

All 4 exon-exon junctions that were found not to be strain-specific had a read coverage of 3, suggesting that, for this study, exon-exon junctions with a read coverage of 3 or less are below the level of reliable detection across the strains and that a higher exon-exon read coverage threshold should be used to confidently identify SSS events. These results, in conjunction with the observation that the median exon-exon read coverage across all strains was ~7.5, led us to define a minimum read coverage threshold of 10. This conservative threshold was defined in an attempt to increase our confidence in identified SSS events. Thus, only potential strain-specific exon-exon junctions with ≥10 read coverage were further analyzed.

### Step 3: reducing potential alignment artifacts

Alignment artifacts, resulting from incorrect alignments of the RNA-seq reads to the genome, have a great potential of negatively impacting our identification of SSS events. Sequencing errors, choice in alignment algorithm, and choice in reference genome used, all have the potential of contributing to alignment artifacts. Of particular concern for this study is that the gapped alignment of the spliced reads defining potential SSS events are the result of alignment artifacts rather than alignments to true exon-exon junctions, particularly if the spliced reads mapped across a non-canonical splice junction. From the list of non-B6 potential SSS events with ≥10 read coverage, we found that approximately 50% had non-canonical splice junctions. To reduce potential alignment artifacts, particularly within non-B6 strains, all potential SSS events defined by non-canonical splice sites were excluded from further analysis.

### Criteria for the identification of high confident strain-specific splicing events

From our protocol, high-confidence SSS events were defined by exon-exon junctions that 1) were uniquely found in one strain, 2) had a read coverage ≥ 10 and 3) were defined by a canonical splice site.

It is important to note that a number of the SSS events that were experimentally validated (Figure 2 and Additional file 1: Table S1) did not meet these criteria. Thus we recognize that these criteria are conservative. Even with these conservative criteria, however, a large number of high-confidence SSS events were observed within each strain (Table 5). NOD and NZO demonstrated the lowest numbers, 40 and 42, respectively, while PWK and CAST demonstrated the highest, 482 and 651, respectively. Please refer to Additional file 2: Table S2 for the genomic coordinates (mm9) of all high-confidence SSS events.

**Table 5:** **High-confidence strain-specific junction statistics for each of the 8 collaborative cross founder strains**

**Strain**	**Total Junctions**	**Annotated Junctions**	**GT/AG**	**GC/AG**	**AT/AC**	Overlap Gene*
129	51	8	47	4	0	32
AJ	53	6	45	7	1	40
B6	66	24	64	2	0	55
CAST	651	18	545	99	7	427
NOD	40	2	32	7	1	22
NZO	42	0	38	4	0	30
PWK	482	23	401	73	8	318
WSB	124	2	109	13	2	76

*Required to be on the same strand.

### The majority of high-confidence strain-specific splicing is Not Yet annotated

Analysis of the high-confidence SSS revealed that an overwhelming majority fall outside of previously annotated exon-exon junctions (Ensembl Version 66) (Table 5). Surprisingly, this was also true of the reference strain, B6, although this strain had the highest percentage of annotated strain-specific exon-exon junctions. Sixty-four percent of the high-confidence SSS events within B6 were unannotated while approximately 96% of the high-confidence SSS events in non-B6 strains have yet to be annotated. It is worth noting that from our set of unannotated SSS, 6 (5 from CAST and 1 from PWK) have been incorporated into a more recent Ensembl (Version 77) annotation while all 13 experimentally validated unannotated exon-exon junctions (Additional file 1: Table S1), in this study, have yet to be incorporated. These results suggest that our current transcriptome annotation of the mouse strains is incomplete, both with respect to the reference B6 strain and to the other founder strains. We also found that the majority of SSS, ~66%, occurred within annotated genes, thus opening up exciting opportunities to further study and understand these splicing events and the specific genes involved.

## Discussion

### Protocol for the identification of high-confidence strain-specific splicing events

Transcript and gene models provide a comprehensive view of our current knowledge of the splicing landscape. However our results show that previously annotated transcript and gene models do not provide a complete view of the splicing landscape, thus for this study, our analysis was focused at the resolution of individual exon-exon junctions and not at the level of individual transcripts and genes. Individual exon-exon junctions are a “transcript model-free” marker of splicing throughout the genome. This granular view allows for an unbiased interrogation of splicing, particularly within regions of the genome not previously annotated or not yet fully characterized. With the ultimate goal of understanding and characterizing SSS within the CC founder strains, it is vital to have a set of splicing events we are confident is specific to a strain thus we identified a set of high-confidence SSS events. Prior to this work, there had not yet been an examination of the criteria needed to define and identify high-confidence SSS events. The identification of SSS using RNA-seq is complicated by both experimental and analytical challenges.

Experimentally, we are limited by the lower representation of reads that span exon-exon junctions (spliced reads) compared with non-spliced reads; furthermore, differential expression of genes and transcripts leads to differential coverage of expressed exon-exon junctions. Thus, even if an exon-exon junction is expressed, it may not be represented among the RNA-seq reads, particularly when its expression is low. Unrepresented but expressed exon-exon junctions within one or more strains may result in false-positive identifications of SSS events. Experimentally, we also need to be cautious of potential sequencing errors. Incorrectly aligned spliced reads, due to sequencing errors within the read, also have the potential of introducing false-positive identifications of SSS events, particularly when the sequencing error occurs in only 1 strain. It is important to note that a portion of the potential strain specific exon-exon junctions identified in Table 4 may have resulted from sequencing errors, particular those with low read coverage. Experimental design, such as separately sequencing the individual samples from each strain; either through barcoding of the individual samples if run on the same lane or having each on a separate lane, rather than pooling the samples before sequencing, can provide support in detecting both sequencing errors and biological noise, and should be considered for future studies. Due to these experimental limitations, the minimum read coverage required to robustly identify an expressed exon-exon junction needed to be determined, noting that this threshold is different for each experiment.

Analytically, we are challenged with accurately aligning RNA-seq reads of the individual CC founder strains to the B6 reference genome. Although the use of genomic scaffolds [25] or imputed genomes [28] for the individual strains could have improved the RNA-seq alignments, the resultant aligned genomic coordinates would have been incompatible thus rendering it virtually impossible to identify conserved and SSS events across the strains. The comparable RNA-seq read mapping percentages of the B6 and the non-B6 strains (Table 1), suggests that near-optimum alignment for each strain was achieved using the B6 reference genome; however great care must still be taken to reduce or avoid potential alignment artifacts stemming from aligning non-B6 RNA-seq reads to the B6 genome.

In this study, the minimum read coverage needed to robustly identify an expressed exon-exon junction was determined using information derived from the experimental validation of a select group of potential strain-specific exon-exon junctions and the overall median exon-exon junction read coverage, while potential alignment artifacts were reduced by focusing on only canonical splice sites. Due to the exon-exon junction focus nature of this study, the expression of individual transcripts and genes This protocol can serve as initial guidelines for the identification of high-confidence SSS events across multiple mouse strains using RNA-seq.

### Utility of strain-specific splicing annotations

The SSS annotations obtained in this study will enhance our understanding and utility of the individual founder strains and all resultant crosses from these strains. Described below are a few examples.

### Implications for insight into phenotypes/disease models

Our focus on the CC and associated outbred populations [39,40] and the use of the striatum as the tissue source for the current experiments stems, in part, from our interest in using these populations to dissect complex behavioral traits [45,46]. From this perspective, many of the SSS events were of considerable interest, in particular because these events may add insight into previously reported strain-specific phenotypes [32-34] and/or models of human disease. For example, the experimentally validated unannotated B6 SSS event within the *Gabra2* subunit (Figure 2) results in the skipping of an exon and is associated with a novel transcript not previously reported in any other species. The function of this truncated transcript, if any, is unknown. However, it is well known that alcohol and benzodiazepines potentiate GABA receptors containing the *a2* subunit, particularly within the B6 strain compared to other laboratory inbred strains, consume excessive quantities of alcohol *e.g*. >20 g/kg/day [47]. Furthermore there is considerable evidence that polymorphisms in the *Gabra2* subunit are associated with alcohol use disorder ([48] and references therein). Overall, these data lead to an easily testable hypothesis. Once the CC recombinant inbred strains are completed and phenotyped, we can test whether this B6 SSS event is significantly associated with those strains showing high alcohol consumption and preference. An alternative approach would be to determine whether this SSS event is segregated with selection for excessive ethanol consumption from any outbred population where the B6 was a founder strain (e.g. the HS-CC).

This same general approach could be applied to any SSS event where the strain is associated with a unique phenotype and there is a reasonable link between the associated biology and the alternative splicing event. High-confidence SSS events found within the non-obese diabetic mouse (NOD) help to further illustrate this point. NOD SSS events were detected in the Fc receptor, IgG high affinity 1 (*Fcgr1*) gene. These events result in: 1) an insertion of a 3 bp exon between exons 3 and 4; 2) a 12 bp insertion in exon 5; and 3) a 4 bp deletion in exon 6 (the last exon). Using Sanger sequencing for 35 mouse strains, it was found that the *Fcgr1* splicing events described above are found only in NOD and closely related strains [49]. Our data confirm the uniqueness of these SSS events and extend the analysis to the WSB and PWK wild-derived strains. Originally, the *Fcgr1* gene was thought to be associated with the autoimmune Type 1 diabetes that develops in the NOD; however, this was subsequently shown not to be the case [50]. More recently the NOD mouse has served as a model to investigate the role of the immune system and activated microglia in a variety of behavioral disturbances. For example, the NOD microglia shows a prolonged reaction to LPS stimulation [51]. These data are consistent with the observation that the NOD mouse reacts with excessive depression-like behavior in response to LPS stimulation [52] which in part may act through an increased influx of IgG into the brain [53]. The precise role of the *Fcgr1* SSS events in the NOD’s excessive physiological and behavioral response is unknown but could be easily tested.

### Insight into strain-specific therapeutic models

Knowledge of SSS may also guide the use of specific mouse strains to the study of specific therapeutics, particularly alternative-splicing-based therapeutics (ASBTs) [35,36]. ASBTs consist of the development of agents (e.g., small molecules, antisense oligos) to restore or correct splicing specific defects associated with a disease. A recent study reported on the potential of two separate small molecules to reverse and induce splicing defects associated with Myotonic Distrophy Type I [36]. In addition, Hua et al. (2011) [35] demonstrated that systemic injection of a chemically modified antisense oligonucleotide specifically targeted to restore the aberrant skipping of exon 7 in the SMN2 gene profoundly ameliorated the viability and phenotypic features of mice affected by a severe form of Spinal Muscular Atrophy (SMA), a neurodegenerative disease [35]. Understanding SSS may allow specific mouse strains to serve as ready-made platforms for the testing and development of ASBTs.

### Improved imputed transcriptomes/genomes for mouse resources

Concerns have been raised regarding the use of the B6 reference genome for aligning RNA-Seq reads from genetically diverse mouse populations. Genomic sequence and structural variations among the different mouse strains were found to cause mapping discrepancies when mapped to the B6 reference transcriptome, particularly within pseudogenes [28]. To decrease these mapping discrepancies, the use of imputed transcriptomes and imputed genomes for individual strains and resultant crosses has been suggested [28,54]. Imputed transcriptomes/genomes utilize annotated genomic variations from the different strains to impute specific transcriptomes/genomes. Although such an approach has the potential to improve the mapping accuracy of RNA-seq reads for non-B6 mouse strains, compared with using the B6 reference, it is severely limited by and dependent on the accuracy and coverage of within-strain genomic variations. It is further limited by the lack of transcriptome annotation for individual strains. Strain-specific splicing annotation in conjunction with previously reported strain-specific genomic variations [25,26] will provide the scaffold needed to further refine imputed genomes and transcriptomes of different mouse resources.

Additionally, our SSS annotation can provide valuable insight into the transcriptome architecture of CC founder strain resultant crosses [19,39,40]. For example, a recent study observed selection biases within CC populations [38], including a number of positively and negatively selected genomic regions. The two most striking were 1) significant over-representation of a 51.6 Mb spanning region of chromosome 2 (73.25–124.85 Mb) from WSB and 2) significant under-representation of a 100 Mb span across chromosome X (35–135 Mb) from CAST. Interestingly, we found SSS within both the over-represented region from WSB and the under-represented region from CAST. A total of 7 and 8 high-confidence SSS events were observed within the positively and negatively selected genomic regions of WSB and CAST, respectively.

## Conclusions

By leveraging the high levels and diverse types of splicing in the brain, this study presents the first comprehensive view of the splicing landscape within the striatum of the 8 CC founder mouse strains. While the majority of the splicing landscape is conserved across the strains, SSS was found within each strain, highlighting the distinct transcriptome architecture of each strain. Two wild-derived strains, CAST and PWK, showed the largest number of SSS events and the most divergent transcriptome architectures. The majority of SSS was found to be not yet annotated suggesting that our current transcriptome annotation of the mouse strains is incomplete, both with respect to the reference B6 strain and to the other founder strains. Interestingly, SSS was also found within genomic regions recently reported to be over- and under-represented within CC populations. Collectively, the SSS information described here provides critical guidance for both transcriptomic and systems genetics analyses in the individual CC founders and its resultant crosses.

### Availability of supporting data

RNA-seq reads for each strain is available in the NCBI Sequence Read Archive (SRA) (http://www.ncbi.nlm.nih.gov/sra) under the following Project ID: PRJNA228935.

## Additional files

Additional file 1:
**List of experimentally validated strain-specific exon-exon junctions.**
Additional file 2:
**Genomic coordinates (mm9) of high-confidence strain-specific splicing events for each CC founder strain.**

## References

[1] Magen I, Chesselet MF (2011). Mouse models of cognitive deficits due to alpha-synuclein pathology. J Parkinsons Dis.

[2] Paumier KL, Sukoff Rizzo SJ, Berger Z, Chen Y, Gonzales C, Kaftan E, Li L, Lotarski S, Monaghan M, Shen W, Stolyar P, Vasilyev D, Zaleska M, D Hirst W, Dunlop J (2013). Behavioral Characterization of A53T Mice Reveals Early and Late Stage Deficits Related to Parkinson’s Disease. PLoS One.

[3] Brown AS, van Driel IR, Hartland EL (2013). Mouse models of Legionnaires' disease. Curr Top Microbiol Immunol..

[4] Lee CY, Cantle JP, Yang XW (2013). Genetic manipulations of mutant huntingtin in mice: new insights into Huntington’s disease pathogenesis. FEBS J.

[5] Moser JM, Bigini P, Schmitt-John T (2013). The wobbler mouse, an ALS animal model. Mol Genet Genomics.

[6] Kwon MC, Berns A (2013). Mouse models for lung cancer. Mol Oncol.

[7] Mucenski ML, Taylor BA, Jenkins NA, Copeland NG (1986). AKXD recombinant inbred strains: models for studying the molecular genetic basis of murine lymphomas. Mol Cell Biol.

[8] Gilbert DJ, Neumann PE, Taylor BA, Jenkins NA, Copeland NG (1993). Susceptibility of AKXD recombinant inbred mouse strains to lymphomas. J Virol.

[9] Ferguson VL, Ayers RA, Bateman TA, Simske SJ (2003). Bone development and age-related bone loss in male C57BL/6J mice. Bone.

[10] Halloran BP, Ferguson VL, Simske SJ, Burghardt A, Venton LL, Majumdar S (2002). Changes in bone structure and mass with advancing age in the male C57BL/6J mouse. J Bone Miner Res.

[11] Anderson MS, Bluestone JA (2005). The NOD mouse: a model of immune dysregulation. Annu Rev Immunol.

[12] Lestaevel P, Airault F, Racine R, Bensoussan H, Dhieux B, Delissen O (2014). Influence of Environmental Enrichment and Depleted Uranium on Behaviour, Cholesterol and Acetylcholine in Apolipoprotein E-Deficient Mice. J Mol Neurosci.

[13] Menke DB (2013). Engineering subtle targeted mutations into the mouse genome. Genesis.

[14] Lynch VJ (2009). Use with caution: developmental systems divergence and potential pitfalls of animal models. Yale J Biol Med.

[15] Mestas J, Hughes CC (2004). Of mice and not men: differences between mouse and human immunology. J Immunol.

[16] Seok J, Warren HS, Cuenca AG, Mindrinos MN, Baker HV, Xu W, Richards DR, McDonald-Smith GP, Gao H, Hennessy L, Finnerty CC, Lopez CM, Honari S, Moore EE, Minei JP, Cuschieri J, Bankey PE, Johnson JL, Sperry J, Nathens AB, Billiar TR, West MA, Jeschke MG, Klein MB, Gamelli RL, Gibran NS, Brownstein BH, Miller-Graziano C, Calvano SE, Mason PH, Cobb JP, Rahme LG, Lowry SF, Maier RV, Moldawer LL, Herndon DN, Davis RW, Xiao W, Tompkins RG (2013). Genomic responses in mouse models poorly mimic human inflammatory diseases. Proc Natl Acad Sci U S A.

[17] Osterburg AR, Hexley P, Supp DM, Robinson CT, Noel G, Ogle C (2013). Concerns over interspecies transcriptional comparisons in mice and humans after trauma. Proc Natl Acad Sci U S A.

[18] Drake AC (2013). Of mice and men: what rodent models don’t tell us. Cell Mol Immunol.

[19] Churchill GA, Airey DC, Allayee H, Angel JM, Attie AD, Beatty J, Beavis WD, Belknap JK, Bennett B, Berrettini W, Bleich A, Bogue M, Broman KW, Buck KJ, Buckler E, Burmeister M, Chesler EJ, Cheverud JM, Clapcote S, Cook MN, Cox RD, Crabbe JC, Crusio WE, Darvasi A, Deschepper CF, Doerge RW, Farber CR, Forejt J, Gaile D, Garlow SJ, Geiger H, Gershenfeld H, Gordon T, Gu J, Gu W, de Haan G, Hayes NL, Heller C, Himmelbauer H, Hitzemann R, Hunter K, Hsu HC, Iraqi FA, Ivandic B, Jacob HJ, Jansen RC, Jepsen KJ, Johnson DK, Johnson TE, Kempermann G, Kendziorski C, Kotb M, Kooy RF, Llamas B, Lammert F, Lassalle JM, Lowenstein PR, Lu L, Lusis A, Manly KF, Marcucio R, Matthews D, Medrano JF, Miller DR, Mittleman G, Mock BA, Mogil JS, Montagutelli X, Morahan G, Morris DG, Mott R, Nadeau JH, Nagase H, Nowakowski RS, O’Hara BF, Osadchuk AV, Page GP, Paigen B, Paigen K, Palmer AA, Pan HJ, Peltonen-Palotie L, Peirce J, Pomp D, Pravenec M, Prows DR, Qi Z, Reeves RH, Roder J, Rosen GD, Schadt EE, Schalkwyk LC, Seltzer Z, Shimomura K, Shou S, Sillanpaa MJ, Siracusa LD, Snoeck HW, Spearow JL, Svenson K, Tarantino LM, Threadgill D, Toth LA, Valdar W, de Villena FP, Warden C, Whatley S, Williams RW, Wiltshire T, Yi N, Zhang D, Zhang M, Zou F, Complex Trait Consortium (2004). The Collaborative Cross, a community resource for the genetic analysis of complex traits. Nat Genet.

[20] Thaisz J, Tsaih SW, Feng M, Philip VM, Zhang Y, Yanas L, Sheehan S, Xu L, Miller DR, Paigen B, Chesler EJ, Churchill GA, Dipetrillo K (2012). Genetic analysis of albuminuria in collaborative cross and multiple mouse intercross populations. Am J Physiol Renal Physiol.

[21] Kelada SN, Aylor DL, Peck BC, Ryan JF, Tavarez U, Buus RJ, Miller DR, Chesler EJ, Threadgill DW, Churchill GA, Pardo-Manuel de Villena F, Collins FS (2012). Genetic analysis of hematological parameters in incipient lines of the collaborative cross. G3 (Bethesda).

[22] Shusterman A, Salyma Y, Nashef A, Soller M, Wilensky A, Mott R, Weiss EI, Houri-Haddad Y, Iraqi FA (2013). Genotype is an important determinant factor of host susceptibility to periodontitis in the Collaborative Cross and inbred mouse populations. BMC Genet.

[23] Ferris MT, Aylor DL, Bottomly D, Whitmore AC, Aicher LD, Bell TA, Bradel-Tretheway B, Bryan JT, Buus RJ, Gralinski LE, Haagmans BL, McMillan L, Miller DR, Rosenzweig E, Valdar W, Wang J, Churchill GA, Threadgill DW, McWeeney SK, Katze MG, Pardo-Manuel de Villena F, Baric RS, Heise MT (2013). Modeling host genetic regulation of influenza pathogenesis in the collaborative cross. PLoS Pathog.

[24] Bottomly D, Ferris MT, Aicher LD, Rosenzweig E, Whitmore A, Aylor DL, Haagmans BL, Gralinski LE, Bradel-Tretheway BG, Bryan JT, Threadgill DW, de Villena FP, Baric RS, Katze MG, Heise M, McWeeney SK (2012). Expression quantitative trait Loci for extreme host response to influenza a in pre-collaborative cross mice. G3 (Bethesda).

[25] Keane TM, Goodstadt L, Danecek P, White MA, Wong K, Yalcin B, Heger A, Agam A, Slater G, Goodson M, Furlotte NA, Eskin E, Nellaker C, Whitley H, Cleak J, Janowitz D, Hernandez-Pliego P, Edwards A, Belgard TG, Oliver PL, McIntyre RE, Bhomra A, Nicod J, Gan X, Yuan W, van der Weyden L, Steward CA, Bala S, Stalker J, Mott R, Durbin R, Jackson IJ, Czechanski A, Guerra-Assuncao JA, Donahue LR, Reinholdt LG, Payseur BA, Ponting CP, Birney E, Flint J, Adams DJ (2011). Mouse genomic variation and its effect on phenotypes and gene regulation. Nature.

[26] Yang H, Wang JR, Didion JP, Buus RJ, Bell TA, Welsh CE, Bonhomme F, Yu AH, Nachman MW, Pialek J, Tucker P, Boursot P, McMillan L, Churchill GA, de Villena FP (2011). Subspecific origin and haplotype diversity in the laboratory mouse. Nat Genet.

[27] Vered K, Durrant C, Mott R, Iraqi FA (2014). Susceptibility to klebsiella pneumonaie infection in collaborative cross mice is a complex trait controlled by at least three loci acting at different time points. BMC Genomics.

[28] Munger SC, Raghupathy N, Choi K, Simons AK, Gatti DM, Hinerfeld DA, Svenson KL, Keller MP, Attie AD, Hibbs MA, Graber JH, Chesler EJ, Churchill GA (2014). RNA-Seq alignment to individualized genomes improves transcript abundance estimates in multiparent populations. Genetics.

[29] Laderas TG, Walter NA, Mooney M, Vartanian K, Darakjian P, Buck K, Harrington CA, Belknap J, Hitzemann R, McWeeney SK (2011). Computational detection of alternative exon usage. Front Neurosci.

[30] Li J, Hakata Y, Takeda E, Liu Q, Iwatani Y, Kozak CA, Miyazawa M (2012). Two genetic determinants acquired late in mus evolution regulate the inclusion of exon 5, which alters mouse APOBEC3 translation efficiency. PLoS Pathog.

[31] Xiong H, Morrison J, Ferris MT, Gralinski LE, Whitmore AC, Green R, Thomas MJ, Tisoncik-Go J, Schroth GP, Pardo-Manuel de Villena F, Baric RS, Heise MT, Peng X, Katze MG (2014). Genomic profiling of collaborative cross founder mice infected with respiratory viruses reveals novel transcripts and infection-related strain-specific gene and isoform expression. G3 (Bethesda).

[32] Flores CA, Cid LP, Sepulveda FV (2010). Strain-dependent differences in electrogenic secretion of electrolytes across mouse colon epithelium. Exp Physiol.

[33] Miller BH, Schultz LE, Gulati A, Su AI, Pletcher MT (2010). Phenotypic characterization of a genetically diverse panel of mice for behavioral despair and anxiety. PLoS One.

[34] Thiesse J, Namati E, Sieren JC, Smith AR, Reinhardt JM, Hoffman EA, McLennan G (2010). Lung structure phenotype variation in inbred mouse strains revealed through in vivo micro-CT imaging. J Appl Physiol.

[35] Hua Y, Sahashi K, Rigo F, Hung G, Horev G, Bennett CF, Krainer AR (2011). Peripheral SMN restoration is essential for long-term rescue of a severe spinal muscular atrophy mouse model. Nature.

[36] Childs-Disney JL, Stepniak-Konieczna E, Tran T, Yildirim I, Park H, Chen CZ, Hoskins J, Southall N, Marugan JJ, Patnaik S, Zheng W, Austin CP, Schatz GC, Sobczak K, Thornton CA, Disney MD (2013). Induction and reversal of myotonic dystrophy type 1 pre-mRNA splicing defects by small molecules. Nat Commun.

[37] Johnson MB, Kawasawa YI, Mason CE, Krsnik Z, Coppola G, Bogdanovic D, Geschwind DH, Mane SM, State MW, Sestan N (2009). Functional and evolutionary insights into human brain development through global transcriptome analysis. Neuron.

[38] Collaborative Cross Consortium (2012). The genome architecture of the Collaborative Cross mouse genetic reference population. Genetics.

[39] Iancu OD, Darakjian P, Walter NA, Malmanger B, Oberbeck D, Belknap J, McWeeney S, Hitzemann R (2010). Genetic diversity and striatal gene networks: focus on the heterogeneous stock-collaborative cross (HS-CC) mouse. BMC Genomics.

[40] Churchill GA, Gatti DM, Munger SC, Svenson KL (2012). The Diversity Outbred mouse population. Mamm Genome.

[41] Dobin A, Davis CA, Schlesinger F, Drenkow J, Zaleski C, Jha S, Batut P, Chaisson M, Gingeras TR (2013). STAR: ultrafast universal RNA-seq aligner. Bioinformatics.

[42] Untergasser A, Cutcutache I, Koressaar T, Ye J, Faircloth BC, Remm M, Rozen SG (2012). Primer3–new capabilities and interfaces. Nucleic Acids Res.

[43] Threadgill DW, Miller DR, Churchill GA, de Villena FP (2011). The collaborative cross: a recombinant inbred mouse population for the systems genetic era. ILAR J.

[44] Gelly JC, Lin HY, de Brevern AG, Chuang TJ, Chen FC (2012). Selective constraint on human pre-mRNA splicing by protein structural properties. Genome Biol Evol.

[45] Iancu OD, Darakjian P, Malmanger B, Walter NA, McWeeney S, Hitzemann R (2012). Gene networks and haloperidol-induced catalepsy. Genes Brain Behav.

[46] Iancu OD, Oberbeck D, Darakjian P, Metten P, McWeeney S, Crabbe JC, Hitzemann R (2013). Selection for drinking in the dark alters brain gene coexpression networks. Alcohol Clin Exp Res.

[47] Yoneyama N, Crabbe JC, Ford MM, Murillo A, Finn DA (2008). Voluntary ethanol consumption in 22 inbred mouse strains. Alcohol.

[48] Li D, Sulovari A, Cheng C, Zhao H, Kranzler HR, Gelernter J (2014). Association of Gamma-Aminobutyric Acid A Receptor alpha 2 Gene (GABRA2) with Alcohol Use Disorder. Neuropsychopharmacology.

[49] Gavin AL, Leiter EH, Hogarth PM (2000). Mouse FcgammaRI: identification and functional characterization of five new alleles. Immunogenetics.

[50] Podolin PL, Denny P, Lord CJ, Hill NJ, Todd JA, Peterson LB, Wicker LS, Lyons PA (1997). Congenic mapping of the insulin-dependent diabetes (Idd) gene, Idd10, localizes two genes mediating the Idd10 effect and eliminates the candidate Fcgr1. J Immunol.

[51] Beumer W, Gibney SM, Drexhage RC, Pont-Lezica L, Doorduin J, Klein HC, Steiner J, Connor TJ, Harkin A, Versnel MA, Drexhage HA (2012). The immune theory of psychiatric diseases: a key role for activated microglia and circulating monocytes. J Leukoc Biol.

[52] Jafarian-Tehrani M, Michaud B, Haour F, Dantzer R, Homo-Delarche F, Bluth (1999). Increased sensitivity of prediabetic nonobese diabetic mouse to the behavioral effects of IL-1. Brain Behav Immun.

[53] Menachem A, Chapman J, Deri Y, Pick CG, Katzav A (2013). Immunoglobulin-mediated neuro-cognitive impairment: new data and a comprehensive review. Clin Rev Allergy Immunol.

[54] Wang JR, de Villena FP, Lawson HA, Cheverud JM, Churchill GA, McMillan L (2012). Imputation of single-nucleotide polymorphisms in inbred mice using local phylogeny. Genetics.

